# DNA Sequence Analysis in Clinical Medicine, Proceeding Cautiously

**DOI:** 10.3389/fmolb.2017.00024

**Published:** 2017-05-03

**Authors:** Moyra Smith

**Affiliations:** Genetics and Genomic Medicine, Pediatrics, School of Medicine, University of CaliforniaIrvine, CA, USA

**Keywords:** nucleic acid sequencing, rare diseases, common diseases, pharmacogenetics, cancer diagnosis

## Abstract

Delineation of underlying genomic and genetic factors in a specific disease may be valuable in establishing a definitive diagnosis and may guide patient management and counseling. In addition, genetic information may be useful in identification of at risk family members. Gene mapping and initial genome sequencing data enabled the development of microarrays to analyze genomic variants. The goal of this review is to consider different generations of sequencing techniques and their application to exome sequencing and whole genome sequencing and their clinical applications. In recent decades, exome sequencing has primarily been used in patient studies. Discussed in some detail, are important measures that have been developed to standardize variant calling and to assess pathogenicity of variants. Examples of cases where exome sequencing has facilitated diagnosis and led to improved medical management are presented. Whole genome sequencing and its clinical relevance are presented particularly in the context of analysis of nucleotide and structural genomic variants in large population studies and in certain patient cohorts. Applications involving analysis of cell free DNA in maternal blood for prenatal diagnosis of specific autosomal trisomies are reviewed. Applications of DNA sequencing to diagnosis and therapeutics of cancer are presented. Also discussed are important recent diagnostic applications of DNA sequencing in cancer, including analysis of tumor derived cell free DNA and exosomes that are present in body fluids. Insights gained into underlying pathogenetic mechanisms of certain complex common diseases, including schizophrenia, macular degeneration, neurodegenerative disease are presented. The relevance of different types of variants, rare, uncommon, and common to disease pathogenesis, and the continuum of causality, are addressed. Pharmogenetic variants detected by DNA sequence analysis are gaining in importance and are particularly relevant to personalized and precision medicine.

## Introduction

Delineation of underlying genomic and genetic factors in a specific disease may be valuable in establishing a definitive diagnosis and may guide patient management and counseling. In addition, genetic information may be useful in identification of at risk family members.

Through international efforts in the Human Genome Project (Lander et al., 2001) a reference sequence of the human genome was derived. In addition, the Human Genome Project facilitated the development of technologies for high throughput sequencing of DNA and enhanced methods for DNA sequence analysis. Capabilities for characterizing human DNA sequence variations, including sequence variations that led to human diseases were thus facilitated.

Availability of the reference human genome sequence enabled the generation of oligonucleotide probes and short sequence markers (SNPs) mapped to specific chromosome positions that could then be used to generate microarray platforms to analyze DNA from specific patients to search for genomic copy number variants and genomic structural variants. Polymorphic SNP markers also facilitated analysis of segregation of specific genomic regions in families and analysis of inheritance of specific genomic regions.

The arrays of DNA based tests currently available for diagnosis of human genetic disease include tests designed to search for mutations in a single gene, gene panel tests designed to search for disease causing mutations in any one of a number of genes that are known to be mutated in a specific type of disease, e.g., diseases associated with ataxia. In addition, second generation sequencing techniques have facilitated analysis of the entire exome or the entire genome in a particular individual.

Through collection of data from large numbers of healthy individuals in populations and through archiving of data in publications, bioinformatics resources have been established that facilitate assessment of the likelihood that a particular genomic variant or sequence variant found in a particular patient is of pathologic significance.

## Different generations of sequencing techniques

First generation sequencing techniques include the sequencing technique described by Maxam and Gilbert (1977) and the dideoxy sequencing technique of Sanger et al. (1977). Second generation sequencing technologies include massively parallel sequencing of gene short read lengths of DNA. The short length sequences then require extensive assembly (Shendure and Ji, 2008). Complex regions of the genome, e.g., regions with high content of repeat sequences present difficulties in assembly.

Third generation sequencing methodologies involve single molecule real-time sequencing (SMRT). This technology was first developed and marketed by Pacific Biosciences (Pac Bio). Pac Bio sequencing has been shown to be particularly useful for sequencing through extended repetitive regions in the genome (Rhoads and Au, 2015). In RNA sequencing, the Pac Bio method has proven valuable for documentation and analysis of multiple mRNA isoforms derived from a single gene. Pac Bio sequencing is also useful in identification of epigenetic modifications of DNA. In epigenetic analysis with the Pac Bio system the DNA does not need to be treated with bisulfite prior to sequencing. Disadvantages of the PC Bio technique include low output and high error rate.

Rhoads and Au noted that for Pac Bio sequencing circular DNA molecules are formed from double stranded DNA using adapters. In each sequencing unit a single molecule of polymerase is immobilized. Replication of DNA is recorded by light pulses and these are individually recorded. Both strands of the DNA molecule are sequenced multiple times. In the first versions of Pac Bio sequence length reads were between 10 and 60 kb.

In human genomics Pac Bio sequencing has been useful in closing gaps in the reference sequence. Pac Bio sequencing has also been useful in characterizing structural genomic variants. A specific sequence analysis method that has proven useful for evaluating structural genomic changes is Parliament that integrates data from short read and long read sequencing (English et al., 2015).

Other long read sequencing technologies that have proven useful in analysis of structural genomic variants include the BioNano Irys system and Illumina Nextera. In the BioNano Irys system a specific enzyme NtBspQ1 nicks long stretches of stained DNA at specific cleavage sites. Nano channel electrophoresis is then carried out to separate DNA fragments. The nickase enzyme digestion followed by Nano channel electrophoresis creates patterns of DNA fragments that can be assembled and compared with reference control DNA. Usher et al. (2015) reported use of this system to analyze the extent of copy number variants and structural genomic changes in the region of the amylase gene cluster in human genomic DNA. The BioNano Irys system is also useful for accurate genomic position analysis of chromosome translocation breakpoints.

The Oxford Minion system uses Nanopore technology and DNA bases are identified as they pass through the Nanopore by measuring their electrical conductivity. Methods for preparation of DNA must be designed to achieve isolation of long DNA fragments. The Minion Nanopore system enables generation of sequence data from DNA fragments up to several thousand bases in length (Lu et al., 2016). The output of sequence from the Minion apparatus is a Fast 5 file. A further important feature is the small size of the Minion sequence it is portable. Connection of the Minion sequencer with sequence data to a computer system by means of a USB port, facilitates data analysis. A specific computer software MinKNOW facilitates sequence analysis. The cloud based system Metrichor can also be used for analysis of Minion sequence data.

## Second generation sequencing of exomes

Exome sequencing analysis involves analysis of exons in all coding genes. One problem with exome sequencing is that all exons in a particular gene or genomic region may not be adequately covered.

Targeted analysis of exome sequence can also be carried out. In these studies, the whole exome is sequenced but analysis initially focuses on a subset of genes that are most appropriate for a specific disorder. It is however important to note that all of the possible genes that give rise to a specific genetic condition or clinical syndrome are not currently known.

It is also important to note that repeat sequences are not adequately represented on exome sequencing and in addition exome sequencing may miss deletion of a specific exon in a particular gene.

### Exon capture methods

Key preliminary steps in whole exome sequencing involve the use of techniques to capture exons. Examples of exome capture methods and kits include Agilent sure select, Nimble gene Seqcapt, and Illumina NRCCE. These three kits were compared in a report by García-García et al. (2016). These investigators reported details on the capture of 870 different genes. They reported that 677 genes were captured by all three methods; 193 genes different in the degree to which they were captured. Comparisons of the three systems revealed that 87% of variants were captured by each of the techniques while 13% of the variants were only captured by one or other of the three techniques. The most favorable capture techniques in the study reported by Garcia-Garcia was Agilent sure select version 2.

Whole exome sequencing is primarily used for detection of single nucleotide variants. Insertion-deletion variants are detected though less reliably and small copy number variants cannot be readily detected. Up to the present exome sequencing has primarily been used for clinical diagnostic purposes, increasingly whole genome sequencing is being applied in clinical cases.

### Clinical outcome following exome sequencing

Clinical outcome may include establishing an unequivocal clinical diagnosis, confirmation of likely clinical diagnosis, failure to confirm a clinical diagnosis, or failure to establish a molecular diagnosis.

Insights into the functions of genes found to carry damaging variants may be gained in some cases through use of databases.

In many cases where apparently pathogenic mutations were found the precise function of the gene was not known. In fact, through biological studies in cases of rare disease with specific gene mutation, insights into the functions of genes are gained.

## Defining mutations as pathogenic

It is becoming evident that the most important first step in determining the pathogenicity of a particular nucleotide variant is to ascertain the frequency of that variant in large population databases, e.g., the ExAC database available at the Broad Institute http://exac.broadinstitute.org/ Truly pathogenic mutations are likely to occur at very, very low frequencies in the general population or not to have ben encountered in the general population. The ExAC databases includes results of DNA analyses on ~60,000 individuals. The key observation is to determine whether the frequency of a specific mutation is higher than the frequency of the specific disease in the population. There are concerns that certain populations are not adequately represented in this database. A general criterion that has been adopted is that in order to be considered rare, a specific variant must occur in a frequency of <1% in the general population.

### Challenges of investigating DNA sequence variants in human disease

Databases such as SIFT and POLYPHEN2 can be consulted to determine if a specific nucleotide variant is likely to influence amino acid sequence and impact protein function. Calculations of variant effect are partly based on evolutionary conservation of particular amino acids.

MacArthur et al. (2014) emphasized that evidence from evolutionary studies or from predictions that a specific sequence variant is deleterious or damaging, does not necessarily indicate that that variant is disease causing. Several investigators have emphasized that prediction of pathogenicity on the basis of evolutionary data can be misleading. Some mutant alleles defined as mutant in a particular species, correspond to the wild type alleles in a different species. It is possible that some sequence variants that are evolutionary retained in certain animal species may be pathogenic in the context of the human genome.

Assessment of variant effects through studies on cells or tissues or in model organisms are valuable in determining pathogenicity. It is also important to emphasize that in the body a specific variant may not always be fully penetrant.

Assignment of degree of pathogenicity of DNA sequence variants will continue to be facilitated through ongoing improvement in data bases on frequencies of DNA variants in different populations, and through enhanced sharing of phenotypic and genetic information.

In 2014 in a publication on clinical exome sequencing, Biesecker and Green emphasized that in analyzing validity of a DNA sequence variant, attention must be paid to analytical validity and clinical validity (Biesecker and Green, 2014). Analytical validity relates to confirmation that the variant exists in a particular individual. To address clinical validity requires attention to the following questions:

- Is the variant pathogenic?
- Is the variant responsible for the disease?
- What is the frequency of the variant in the general population?
- Do assessments of clinical databases indicate that this variant has previously been encountered in patients with a similar disease phenotype?

Biesecker and Green emphasized that establishing clinical validity of a sequence variant potentially has a number of positive benefits including ending of a diagnostic odyssey and providing useful information for the patient and the family.

Biesecker and Green noted further that finding of a genetic variant in some cases fueled additional clinical examination, additional history taking or additional special studies. They noted that in the cases where no informative sequence variant is found, it is important to propose ongoing interpretation of data as more knowledge and more data become available.

*In silico* prediction tools have been shown to make errors in certain cases where experimental methods have proved pathogenicity of a particular variant (Azevedo et al., 2016). In addition, examples of miscalling of mutations as pathogenic have been encountered; for example, in reports on pathogenic genes in cardiomyopathy. Walsh et al. (2016) published evidence that 40 of the 60 genes reported previously to be involved in cardiomyopathy, were likely irrelevant.

## American college of medical genetics (ACMG) guidelines for the interpretation of sequence variants

In 2015 the ACMG (Richards et al., 2015) reported guidelines for the interpretation of sequence variants identified in protein coding DNA. They proposed four different levels for classifying mutation as pathogenic: very strong PVS, strong PS1, moderately strong PM, and possibly strong PP. PVS mutations included null, nonsense, or frameshift mutations, mutations that impacted canonical splice sites, mutations that interrupted transcription initiation sites, deletions that included an exon or multiple exons. PS1 mutations included those mutations where the same aminoacid has been found to be mutated in patients affected with similar disease. PM mutations included mutations in a functional domain of the proteins where the mutation has not been reported in the general population. PP mutations include mutations that co-segregate with the disease in multiple family members. Benign mutations were defined as mutations that occur with a frequency >5% in the general population and mutations that occur with higher frequency than the frequency of a specific disease in the general population.

It is important to note that structural changes in in the genome may lead to disease. Furthermore, deletion of a specific exons or of more than one exon may not readily be identified on exome sequencing.

With respect to stop codon mutations it is important to note that there a three different potential stop codons in DNA TAG, TAA, TGA in mRNA UAG UAA, UGA. Stop codons result in release of ribosomes from mRNA. Partially synthesized proteins are then also released from the ribosomes and these may undergo degradation in a nonsense mediated decay process. However, nonsense mediated decay does not result if stop codons are located in the terminal exons of a gene and truncated proteins may then be retained in the cell.

It is important to note that specific missense mutations may also impair protein function. Evers et al. (2017) reported that replacement of leucine by proline disrupted the helical structure of an important domain in the DYRK1A protein that functions as a kinase. Another missense mutation in DYRK1A ARG467 affected the stability of the protein. Specific missense mutation may impair interaction of an enzyme protein with its substrate.

Quintáns et al. (2014) noted that in defining splicing mutations as pathogenic investigators did not always take into account the existence of alternative splicing for most gene products or the evidence for tissue specific splicing.

Additional factors that need to be considered in the follow-up to clinical sequencing include translation of data to referring physicians and communication of data to patients and families. Legal and social implications of sequencing data also need to be considered.

## Application of sequencing to diagnosis of rare diseases likely of genetic origins

It is estimated that the number of rare diseases due to gene defect exceeds 6,800 (https://www.genome.gov). Many families with children with rare diseases experience the diagnostic odyssey that involves multiple specialist visits, laboratory and imaging studies and in some cases surgical procedures. Despite extensive testing in many cases diagnoses are not made. In addition, there is evidence that in cases of rare diseases the initial diagnoses made are incorrect in 40% of cases (Sawyer et al., 2016). In some cases, establishing a correct diagnosis was complicated by unusual clinical presentation. In addition, genetic heterogeneity of disorders where defects in a number of different genes can give rise to the same clinical or syndromic manifestations, complicates reaching a diagnosis. Sawyer et al. noted that Joubert syndrome was a case in point where mutations in any one of 20 different genes lead to the same clinical manifestations. In addition, for many genetic disorders the most characteristic features of that disorder are not present in young infants. Furthermore, defects in a specific gene may lead to presence of different subsets of clinical manifestations in a specific patient.

### Examples of cases where finding a DNA mutation significantly altered medically management

A particularly striking case where exome sequencing revealed correct diagnosis that led to altered case management was reported by Worthey et al. (2011). A 15-month old male child with transmural colitis, colo-cutaneous fistulas, proctitis, and peri-anal abscesses failed to respond to therapy for inflammatory bowel disease. Exome sequencing revealed that the child had a deleterious mutation in the X-linked gene that encodes the inhibitor of apoptosis protein XIAP. Worthey et al. then initiated the recommended treatment for deficiency of X-linked inhibitor of apoptosis, namely allogeneic hematopoietic stem cell transplantation. This treatment led to dramatic improvement in the health of the child.

In 2012, Johnson et al. used genetic linkage analysis and exome sequencing to identify causative mutations in an extended Lebanese family with several members affected with Brown Vialetto Van Laere disease. This disease, first described in 1894, is characterized by motor neuron defects and progressive sensorineural deafness. Johnson et al. identified damaging mutations in the gene *SLC52A3* that encodes a riboflavin transporter RFVT3. Initial riboflavin treatment in one of the affected individuals showed promising results.

In 2014, Foley et al. used exome and Sanger sequencing to search for mutation in 18 patients from 13 families with Brown Vialetto Van Laere disease. Sequencing results revealed the presence of mutations of in *SLC52A2*, compound heterozygous mutations in some of the patients and homozygous deleterious mutations other patients. *SLC52A2* encodes the riboflavin transporter RFVT2. The affected patients in the families they studied had rapidly progressive axonal sensory-motor neuropathy with ataxia, particularly in the upper limbs and axial muscles. In addition, they had hearing loss and optical atrophy. Foley et al. carried out biochemical studies that revealed reduced riboflavin uptake and reduced riboflavin transporter protein expression. They reported that high doses of oral riboflavin led to significant biochemical and clinical improvements in 10 patients.

In 2015, Petrovski et al. reported results of exome sequencing in a 20-month old child with a rapidly progressing neurological disease associated with ataxia, nystagmus and flaccid paralysis of the upper arms and weakness of the neck muscles. This child had originally been diagnosed with an auto-immune condition and was being treated with high doses of steroids. Despite that treatment the child's condition continued to deteriorate. Exome sequencing revealed two loss of function variants in the gene *SLC52A2*, indicating a diagnosis of Brown Vialetto Van Laere disease. The therapeutic management was altered and treatment commenced with riboflavin. Petrovski et al. reported that clinical improvement was soon evident. In 2015, Shashi et al. published a follow-up report on this child and noted that sustained clinical improvement was observed over an 8-month period.

### Identification of molecular defects in dystonias sometimes classified as cerebral palsy

Dopamine responsive dystonias are a group of disorders that often present in childhood and are characterized by abnormal movements. Dystonic movements include hypokinetic and hyperkinetic movements frequently classified as cerebral palsy.

One form of dystonia Segawa disease, is known to be due to defects in the GTP cyclohydrolase encoding gene (*GCH1*) that encodes an enzyme required for neurotransmitter biosynthesis. Torsion dystonia has also been described in cases with tyrosine hydroxylase deficiency.

In 2011, Bainbridge et al. studied a pair of fraternal twins with torsion dystonia where no mutations in GTP cyclohydrolase or tyrosine hydroxylase were present. In addition, the symptoms in these patients were not relieved by L-dopa and carbidopa. Despite therapy these twin suffered dystonia, choreiform movements, and unsteady gait. A particularly difficult problem that emerged was respiratory difficulties due to laryngospasm. Genome sequencing in these twins revealed damaging variants in the SPR gene that encodes sepiapterin reductase an enzyme critical to the synthesis of tetrahydrobiopterin (BH4) a critical cofactor in the synthesis of dopamine and catecholamines. Both affected twins had compound heterozygous mutations in the SPR gene. One mutation was a nonsense mutation, p.Lys251X, inherited from the father and the other was a missense mutation, p. Arg150Gly inherited from the mother. An unaffected older sibling of the twins, had neither of the mutations. Since there was evidence that patients with SPR mutations benefited from treatment with L-Dopa/carbidopa and from treatment with 5-hydroxytryptophan (5HTP) the latter compound was added to the twins' treatment. An important follow-up observation was that addition of 5HTP to treatment led to marked symptom improvements and the laryngospasm abated.

### Definitive molecular diagnosis of a movement disorder with Parkinson disease features in childhood

Rilstone et al. (2013) described an extended consanguineous Saudi-Arabian family with eight children affected with movement disorder. The index case presented with ataxia, tremor, and Parkinsonian type shuffling gait. Results of blood and cerebrospinal neurometabolite studies were normal. However, elevated levels of monoamine derivatives were found in urine. Levels of 5-hydroxyindole acetic acid and homovanillic acid were increased, while levels of urinary dopamine were low. On the basis of the urinary findings and the presence of Parkinsonism features, the proband and three siblings were treated with l-dopa-carbidopa. However, the dystonia and choreiform movements worsened on treatment so that it had to be discontinued.

Genetic and genomic studies undertaken by Rilstone et al. included SNP marker analysis that led to the identification of a 3.2 megabase interval on 10q25.3–10q26.1 that was shared by affected family members. Several genes within this region were then sequenced and a variant was found in the *SLC18A2* gene that encodes a solute carrier protein VAT2 that carries monamines across the synaptic vesicle membrane. The variant in SLC18A2 c.1160C>T, p. P387L was also present on exome sequencing studies in the proband. This variant was not present in the 1,000 genomes database. Rilstone et al. expressed the protein in cells and they demonstrated that the mutant protein had severe loss of function.

In view of the reduced function of the reduced protein, Rilstone et al. initiated treatment with a dopamine receptor agonist and this treatment resulted in dramatic and sustained disappearance of Parkinsonism, dystonia and other symptoms. The investigators noted that in the 32nd month of treatment continuing benefits and minimal side effects were noted.

In 2016, Jacobsen et al. reported that exome sequencing on infant twins with developmental delay, hypotonia and oculogyric crises (prolonged upward deviation of the eyes), revealed a SLC18A2 mutation, the mutation in these infants was p. pro237his. The twins also benefitted from treatment with a dopamine receptor agonist.

### DNA sequencing in analysis of unexplained rare abnormal metabolic phenotypes

Tarailo-Graovac et al. (2016) reported results of the combined use of deep phenotyping, metabolite and biochemical analyses and whole exome sequencing studies in patients with developmental disorders and unexplained metabolic phenotypes.

The investigators defined a metabolic phenotype as a pattern of abnormal metabolites in urine blood or cerebrospinal fluid or abnormal studies at the cellular level e.g., abnormal mitochondrial functional studies. Deep phenotyping included examination for evidence of developmental or cognitive regression, and clinical examination for abnormal features or organ abnormalities. It also included neuroimaging and histopathology to search for abnormal storage vacuoles. DNA from blood or saliva was used for exome sequencing.

The combined analytical approach led to diagnosis of the underlying genetic defect in 28 of 41 patients (68% the patients studied). In 22 cases previously reported gene variants were associated with newly identified phenotypic features. In 2 cases the genetic defects were found in genes not previously reported in disease. Significantly this study led to changes in treatment and to initiation of specific targeted intervention.

## Exome sequencing to establish molecular diagnosis in a heterogeneous genetic disorder: limb girdle muscular dystrophy

In 2015, Ghaoui et al. reported results of exome sequencing on patients with limb girdle muscular dystrophy where conventional studies, including histology, protein studies, and candidate gene sequencing had failed to reveal the cause of the disease. They noted that 27 forms of limb girdle muscular dystrophy are known, including dominant inherited, recessive and X linked forms. Immunohistochemistry studies carried out on their patients, included analysis of the proteins merosin, alpha-dystroglycan, alpha sarcoglycans, gamma sarcoglycans, delta sarcoglycans, dysferlin, caveolin3, desmin, mytotilin, and type VI collagen. Western blotting analysis was carried out for dystrophin alpha dystroglycan, lamin A/C. emerin and calpain 3. In addition, single gene analyses were carried out for genes that encode proteins not analyzed in their protein studies these included the Fukutin and Fukutin related protein genes (*FKTN* and *FKRP*) and the protein mannosyl protein encoding genes (*POMT1* and *POMT2*), the alpha glucosidase encoding gene (*GAA)* and anoctamin (*ANO*), and telethonin (*TCAP*) genes.

Despite this extensive array of testing in 65% of their patients a molecular genetic diagnosis was not made. Exome sequencing was then carried out in 60 of these patients. Molecular diagnosis was made in 27 of the patients. Twelve of the patients were found to carry mutation in genes known to cause limb-girdle muscular dystrophy. In 14 of the patients, mutations were found in genes typically associated with other forms of inherited myopathy. Two patients were found to have metabolic forms of myopathy one due to defects in the muscle glycogen phosphorylase encoding gene (*PYGM*) and the other due to defects in the carnitine palmitoyl transferase 2 gene (*CPT2*). One patient had mutation in the gene that encodes torsin interacting protein (*TOR1AIP1*). Mutations in that gene are typically associated with contractures and cardiomyopathy.

Importantly despite exome sequencing molecular diagnosis was not established in 33 of the 60 patients. (Ghaoui et al., 2015) considered factors that likely contributed to this failure. These included poor capture of specific exons, deletions of exons, presence of repetitive sequence elements.

In 2016, Ghaoui et al. reported discovery of gene mutations in the chaperone encoding gene *HSPB8* in a case of distal myopathy with motor neuropathy. HSPB8 protein contributes to formation of the chaperone assisted selective autophagy complex. Mutations in other members of this complex have been previously reported to be important causes of muscular dystrophy associated with myofibrillar myopathy, presence of aggregates, and rimmed vacuoles in muscle.

In a review of guidelines for diagnosis and treatment of limb girdle and distal muscular dystrophies, Narayanaswami et al. (2014) emphasized that establishing a clinical diagnosis of a specific form of limb girdle muscular dystrophy was difficult since specific forms did not necessarily have specific pathognomonic features.

They particularly emphasized that it was important to distinguish genetic forms of these disorders from acquired myopathies since the latter are often treated with immunosuppressive agents that have significant side effects.

## Whole genome sequencing and exome sequencing to facilitate diagnosis in inborn errors of metabolism

Modification of whole genome sequencing techniques and analyses that led to more rapid turnaround in data generation have been reported by the Kingsmore and Saunders (2011) and by Stranneheim and Wedell (2016). The more rapid turnaround and generation of data are particularly important in young infants. In some cases, appropriate treatment can be then rapidly initiated.

Stranneheim and Wedell (2016) applied DNA sequencing to solve diagnostic problem in cases with suspected inborn errors of metabolism. They described a case with increased methionine levels where normal results were obtained in studies on all four genes known to harbor mutations in cases of hypermethioninemia. In their unsolved case exome sequencing subsequently revealed pathogenic mutation in the *ADK* gene that encodes adenylate kinase. The authors noted that discovery of this mutation and its physiological effect served to link the methionine and adenosine metabolic pathways.

Stranneheim and Wedell emphasized that mitochondrial dysfunction is an important source of inborn errors of metabolism. Effective function of mitochondria is dependent on normal function of 37 products encoded by the mitochondrial genome and on adequate function of more than 1,000 nuclear genes. In addition, coordinated expression of gene in the two compartments is required. Exome sequencing to examine nuclear encoded genes that function in mitochondria and sequencing of the mitochondrial genome are therefore important in defining the genetic cause of inborn errors of metabolism. New gene defects leading to mitochondrial dysfunction have been discovered through exome sequencing.

Stranneheim and Wedell drew attention to the fact that exome sequencing findings have served to expand information on genotype phenotype correlations. For example, there is growing evidence that different mutations in the same gene can give rise to different phenotypes. An example they presented involved mutations in the *AARS2* gene that encodes alanyl-t-RNA synthetase. Mutations in this gene were initially described as giving rise to cardiac dysfunction. Different mutations in *AARS2* were found to give rise to ataxia, spasticity and cognitive defects.

## Comparing microarray data exome sequencing and whole genome sequencing data

Stavropoulos et al. (2016) reviewed results of different types of genomic studies on patients with neurodevelopmental delay. They noted that prior reports revealed that in 80–85% of cases with neurodevelopmental delay a molecular diagnosis could not be established by clinical microarray analyses carried to detect pathologically significant genomic deletions or duplications. Exome sequencing established a molecular diagnosis in only ~2% of patients with neurodevelopment delay and/or congenital malformation.

In the study reported by Stavropoulos et al. whole genome sequencing studies were carried out on patients with the following clinical manifestations; developmental delay, neurological symptoms, skeletal abnormalities, growth defects, eye defects cardiovascular defects, and muscle defects. In their study in each individual genome on average 3.5 million single nucleotide variants and 248 copy number variants occurred, in addition 1,604 smaller structural variants including duplications and deletions were present and 20,014 exonic and splice site variants were present. They noted that 28% of CNVs and 2% of structural variants impacted coding regions. Whole genome analysis permitted analysis of breakpoints of copy number variants.

Key to their data analysis was development of a pipeline that filtered variants to prioritize clinically significant variants. Stavropoulos et al. concluded that molecular diagnosis was established in 66 of 104 patients. The diagnostic yield was higher when trio sequencing (affected individual and parents) was carried out.

Stavropoulos et al. reported that in 17 of 22 cases the molecular diagnostic marker found on whole genome sequencing was different than the gene defect that had been predicted on the basis of clinical findings prior to sequencing.

## Analysis of cell free fetal DNA and non-invasive prenatal diagnosis for trisomy

In 1997, Lo et al. reported that it was possible to isolate fetal DNA from maternal blood. In 2007, Lo and Chiu reported that fetal DNA derived from maternal blood could be used for prenatal diagnosis purposes. In 2008, Chiu et al. reported use of massively parallel genome sequencing for analysis of DNA from maternal plasma. In each case a specific quantity of DNA, 100 ng, was derived from plasma from a maternal plasma. They noted that since the DNA in plasma was already fragmented, no additional fragmentation was required prior to sequencing. Following sequencing of DNA according to massively parallel sequencing protocols, the sequenced fragments were aligned with the reference genome under condition that blocked repetitive sequences in the genome. Along each individual chromosome the number of aligned sequences was determined. The number of reads was determined for each chromosome for tests samples and controls. The numbers of aligned reads per chromosome correlated with the length of the chromosome. Analyses were then carried out to determine if the number of aligned fragments for a specific chromosome in the test sample correlated in test and control samples. Chui reported that genomic sequencing enabled diagnosis of trisomy 21.

In 2008, Fan et al. reported isolation and massively parallel sequencing of DNA from maternal plasma and successful diagnosis of fetal trisomy of chromosomes 21, 18, and 13.

There are ongoing research studies to determine if this technology can be used for detection of other chromosome abnormalities in fetuses e.g., deletions or translocation.

Currently in clinical prenatal diagnosis, DNA sequencing analysis of cell free DNA isolated from maternal plasma is used to screen for trisomy 13, 18, and 21. Other methods are also routinely used to assess risk of fetal abnormalities these include nuchal translucency evaluation, ultra-sound and biochemical marker analysis, including analysis of pregnancy associated protein A, and beta subunit of human chorionic gonadotrophin.

## Pharmacogenomics

In pharmacogenomics emphasis is placed on genetic variants that impact genes that encode proteins and enzymes involved in drug absorption, distribution, drug action, metabolism, and toxicity. Genetic variants may impact the level of gene expression and the specific function of the protein or enzyme.

The Pharmacogenomic knowledge base website https://www.pharmgkb.org (accessed Jan 27th 2017) lists 333 genes with variants that impact drug uptake, metabolism or function. Particularly important in drug metabolism are cytochrome p450 enzymes in particular CYP2D6.

Tables available at the Pharmacogenomic knowledge base website alphabetically list ~331 drugs where therapeutic efficacies are impacted by genetic variant in human. Labels indicating that genetic testing is required prior to use of the specific drug was noted for ~50 drugs.

In 2014, Kampourakis et al. reported that in Europe 150 drugs were reported to require labels with pharmacogenomic and related information including warning of adverse effects.

### Pre-emptive genotyping for clinical pharmacogenetics

Dunnenberger et al. (2015) reviewed the application of microarray testing for genotypes of genes relevant to clinical pharmacology. They emphasized the importance of genotyping of patients prior to the use of high risk drugs and noted that information on clinically actionable pharmacogenetics variants continues to grow.

Initially pharmacogenetics variant testing was done on a gene by gene basis. Currently high quality genotyping testing is available and sites are listed at: https://www.ncbi.nlm.nih.gov/gtr/.

Dunnenberger et al. emphasized that ~1,000 drugs are approved for clinical use by the FDA in the USA and 10% of require pharmacogenetics testing prior to use. These authors noted that genotyping of 12 different genes was particularly important in pharmacogenetics. These genes and the proteins they encode are listed below.

CYP2C19 cytochrome p450 family 2 subfamily C19
CYP2C9 cytochromep450 family 2 subfamily C9
CYP2D6 cytochrome p450 family 2 subfamily D6
CYP3A5 cytochrome p450 family3 subfamily A5
DPYD dihydropyridine dehydrogenase
G6PD glucose-6-phosphate dehydrogenase
HLAB histocompatibility locus B
IFNL3 interferon lambda 3
SLCO1B1 solute carrier organic anion transporter
TPMT thiopurine-S- methyltransferase
UGT1A1 UDP glucuronosyl transferase
VKORC1 Vitamin K epoxide reductase complex.

Population differences exist in genotypes of these genes and these differences can lead to different susceptibilities to adverse drug effects. Dunnenberger et al. noted that variants in *IFNL3* and *CYP3A5* were particularly important in adverse drug effects in African Americans. *VKORC1* variants leading to adverse drug effects were observed primarily in white Americans. Dunnenberger et al. listed the top 30 drugs with highest pharmacogenetics associated adverse risks. Tests of *TPMT* genotypes is particularly important in patients being treated for malignancies. They noted that at St Jude's Research Hospital 48% of pediatric patients received at least one high risk drug per year. Vanderbilt University Medical Center reported that 54% of adult patients were prescribed a high risk drug per year. Drugs associated with adverse effects due to genetic variants are listed at http://www.pharmgkb.org.

Dunnenberger et al. emphasized the importance of having drug sensitivity information available in an easily interpretable form in the patients' electronic medical records.

## Diversity of genetic architecture

Human geneticists have primarily classified genetic diseases as monogenic, oligogenic, or complex disease. In a monogenic disease one particular gene has undergone mutation that leads to a disease. In oligogenic diseases variants at a number of different loci in the genome can influence the phenotype in a specific disease. In complex diseases large numbers of variants, each of small effect, can constitute risk factors for the disease and influence the disease phenotype. Neutral variants that do not lead to disease are also known. In addition, somatic variants can occur throughout life and some of these may constitute cancer causing variants.

Advances in molecular genetics increasingly provide information on regulatory variants that impact phenotypes Genetic variants in non-protein regions of the genome may impact regulation of gene expression. Studies on variants in non- protein coding regions of the genome are actively ongoing. These studies involve analyses on enhancer elements, variants in gene promoters, transcription factor binding sites, studies on chromatin and chromatin modifying factors and epigenetic factors that modify DNA.

## Interplay between different variant types

Lupski et al. (2011) stressed that the unique combination of an individual's mutational burden must be taken into account. They stated that emphasis must be shifted to encompass a whole genome views. The genome of an individual includes inherited alleles and *de novo* variants. An individual genome includes variants that were present in ancient ancestors and in addition an individual has variants that arose more recently in relatives and *de novo* variants. *De novo* variants may have arisen in germ cells or at any stage of development of later life.

In addition, the genome contains a collection of protective variants and deleterious variants that act in combination constituting an ecology. Lupski et al. emphasized that the concept of genome ecology has ramifications that impact the medical utility of genetic information.

Lupski et al. concluded that recent rare variants are likely to be most medically relevant. However, they also noted that in some individuals, unusual combinations of common variants may contribute to disease risk. The severity of the phenotype of a specific disease in a particular individual may be impacted by that individual having mutations in two different genes that are associated with a specific disease. The extent to which heterozygous carriers of specific disease risk alleles have disease manifestations is also of interest.

Specific genes that carry disease inducing mutations may also harbor variants that influence complex diseases. One example they described was the ABCA4 gene that carries a mutation that impacts risk for age related macular degeneration and also carries mutations that predispose to Charcot Marie Tooth neuropathy.

## The continuum of causality

In 2016, Katsanis published an interesting paper on the continuum of causality in genetic diseases. He noted that human geneticists had in the past followed a reductionist paradigm that separated rare disorders and complex disorders. Geneticists separated monogenic and polygenic disorders. He emphasized that strict separation of these two types of genetic disorders and the concept of one gene one phenotype needed to be revised in the light of recent information on genomes and phenotypes. There is now evidence that the phenotype caused by mutation in a specific gene is modified by other genes. The phenotype in some cases may even be modified by regulatory factors or even by allelic variants downstream of that gene but on the same haplotype. An example of the latter is the finding that a polymorphic microsatellite repeat sequence element D4Z4 modulates expression of mutations in the *SMCHD1* gene and modulates the phenotype severity in facioscapulo-humeral muscular dystrophy.

As an example of the continuum between Mendelian disease and complex common diseases, Katsanis noted that specific mutations in the melanocortin receptor gene *MC4R* predispose to severe obesity that follows a Mendelian inheritance pattern. However, other mutation in that gene predispose to a complex disorders characterized by obesity and predisposition to diabetes.

## Genetic variants in cancer

In 2013, Vogelstein et al. reported that 140 genes contained mutations that acted to drive cancer. They noted that not all mutations in each of those genes acted as driver mutation. The driver gene mutation led to growth advantage.

In 2015, Martincorena and Campbell reported that 572 different genes were recurrently mutated in cancer.

In 2017, Li et al. reported standards and guidelines for interpretation and reporting of sequence variants in cancer. This report was based on recommendations of individuals representing molecular pathologists and clinical oncologists. A four tiered system to categorize somatic sequence in tumors was proposed.

Tier I included variants with strong clinical significance. Variants with clinical significance included variants where there was prior evidence of significance for diagnosis, prognosis, or significance.

Tier II included variants with potential clinical significance. Tier III included variants of unknown clinical significance. Tier IV included variants classified as benign.

The authors noted that cancer genomics was rapidly evolving and that it was important not only to have standards for reporting but also to follow up on new information and treatment responses. Important databases that document mutations include the National Cancer Institute database USA, https://gdc.cancer.gov and the UK database http://cancer.sanger.ac.uk/cosmic.

Li et al. noted that *TP53* is the most commonly mutated gene in common cancers and that clinical trials were in place to test compounds with the potential to restore TP53 normal function.

In addition to information on tumor specific genomic variants it is important to determine whether particular tumor pathogenic variants occur in an individual's normal tissues (germline mutations). Germline mutations occur in certain cancers that follow a Mendelian pattern of inheritance. These tumors are sometimes referred to as hereditary or familial cancers and specific databases list these tumors, http://ghr.nlm.nih.gov.

### Genetic alteration in cancer cells that generate neoantigens, implications for immunotherapy

Desrichard et al. (2016) reported progress in efforts to characterize tumor neoantigens. These can be generated from specific nucleotide mutations, including loss of functions mutations, altered reading frames, and fusion transcripts. The neoantigens are altered proteins and peptides. Structures of the neoantigen peptides are being submitted in specific analysis algorithms (e.g., NETMHC) http://www.cbs.dtu.dk/services/NetMHC/ to determine which peptides are particularly immunogenic.

Desrichard et al. noted that only a fraction of patients benefit from immunotherapy. Studies are being designed to determine which patients are likely to respond. They noted that higher mutation burden tends to lead to generation of more immunogenic peptides.

Studies are also in place to determine which cancer specific elements, including peptides can be used as cancer vaccines (Lu and Robbins, 2016).

In 2017, Davoli et al. reported on comparative analysis of numerical chromosome abnormalities, degree of aneuploidy and tumor response to immunotherapy. Their study revealed that in the presence of high chromosome numbers in tumors, there was reduced expression of cytotoxic immune infiltrates in the tumors and reduced response to immunotherapy.

### Sequencing and liquid biopsy in cancer

Tumors have been shown to give rise to cell free DNA that can be detected in body fluids including plasma, cerebrospinal fluids. Sequencing of cell free DNA has been shown to be of value in establishing whether or not therapies are effective (Lebofsky et al., 2015).

In addition, tumors also shed vesicular structures exosomes that contain DNA and also RNA and tumor specific nucleic acid variants can be detected through sequencing. Exosome studies are also proving valuable in cancer diagnosis and in monitoring treatment response and possible occurrence of metastases (Kalluri, 2016).

## Sequence variants in complex common diseases

### DNA sequencing in analysis of schizophrenia

In 2016, Genovese et al. published data on exome sequence analysis carried out in 12,332 unrelated Swedish individuals including 4,877 individuals with schizophrenia. The purpose of their study was to examine the frequency of ultra- rare DNA sequence variants that impacted protein function. The variants included nonsense, frame shift and splice site variants, read-through variants and missense variants, and deletion or insertion variant that impacted protein-protein interactions. Variants were classified as being ultra-rare based on their absence in the general Swedish population and on their absence in the ExAC database of populations variants.

Genovese et al. reported that their data analysis revealed significant differences between cases and controls in the frequencies of disruptive ultra-rare variants (dURVs). They reported that schizophrenia affected individuals carried 7% more dURVs than controls.

Genovese et al. did not directly sequence parents of affected cases in this study. However, based on calculation of the mutation rates of synonymous and non-coding DNA variants, and evidence that these were similar in affected cases and controls, they concluded that the disruptive ultra-rare variants in schizophrenia patients were likely inherited.

The tissue and cellular expression of genes substantially impacted by disruptive ultra- rare variants was examined. Genovese et al. determined that the impacted genes were primarily but not exclusively expressed in brain. Furthermore, within the brain the genes tended to be expressed preferentially in neurons rather than in astrocytes or oligodendrocytes.

A number of the genes implicated in schizophrenia on the basis of the occurrence of disruptive ultra-rare variants overlapped with genes associated X linked intellectual disability genes. Genovese et al. reported that their analyses did not identify ultra-rare variants of large effect.

## Complex common diseases: common, low frequency and rare sequence variants

### Macular degeneration

Age related macular degeneration (AMD) is a significant cause of blindness in the elderly. AMD is associated with accumulations of deposits of proteins, lipids and cellular debris in the extra-cellular matrix beneath the retinal pigment epithelial cells. These deposits, referred to as drusen, impair the supply of nutrients to the retinal photoreceptor cells. Schramm et al. (2014) reported that the formation of drusen deposits precedes development of both forms of AMD the neovascular (wet) form and the atrophic (dry form). Genome wide association studies revealed that variants in genes encoding complement related factors play roles in AMD. Schramm et al. proposed that complement factors play roles in maintaining the balance between activation of complement that facilitates clearance of debris, and mechanism of timely repression of complement function to avoid collateral tissue damage.

In a large international collaborative study, Fritsche et al. (2016) reported results of analysis of common and rare genetic variants in 16,144 AMD patients and in 17,832 controls. They identified high frequency common variants and rare variants in 34 different genes in AMD patients. Eight different gene loci and the proteins they encode, that contained the highest number of variants are listed below:

*FH* complement factor H
*C2/CFB* Two adjacent loci that encode complement C2 and complement factor B
*ARMS* age related maculopathy susceptibility gene 2
*C3* complement 3
*APOE* apolipoprotein E
*CHI* complement factor I (serine proteinase)
*TIMP3* metallopeptidase inhibitor
*CETP* cholesterol ester transfer protein.

Fritsche et al. also identified a variant that was specifically associated with the wet form of macular degeneration. This variant was near to the *MMP9* gene that encodes metallopeptidase 9.

The fact that these particular genes act as risk factors for AMD indicate that the biological pathways implicated in AMD include the alternative complement pathway and the lipid transfer pathway (Fritsche et al., 2016).

## Neurodegenerative diseases, risk factors revealed through genomic variant analysis

### Low frequency variants in the *TREM2* gene in Alzheimer disease

In studies in the Icelandic population, Jonsson and Stefansson (2013) identified that a specific nucleotide variant in the *TREM2* gene. The variant was designated rs75932628. The reference nucleotide in rs75932628 is C and the variant nucleotide is T. The variant nucleotide leads to an amino acid substitution R47H. The variant nucleotide T occurs with a frequency of 0.002 in the population and it is therefore a low frequency variant.

In 2015, Lill et al. reported that in a study of 24,086 cases of Alzheimer disease and 148,493 controls carrier status for the variant form of rs75932628 was associated with increased risk of Alzheimer disease. These authors also reported that carriers of the variant form of rs75932628 had increased levels of the protein Tau.

The TREM2 gene encodes a receptor on microglial cells and likely plays a role in inflammatory responses. Wood (2017) reported that microglial responses and neuroinflammation are implicated in the pathogenesis of Alzheimer disease.

### Genomic variants that impact risk for more than one form of neurodegenerative disease

Genetic studies including genome wide association studies and DNA sequencing studies have revealed that variants in a number of different genes increase risk for development of late onset neurodegenerative diseases. These studies have also revealed that variants in specific genes may constitute risk factors for more than one neurodegenerative disease. One clear example of this is the occurrence of repeat expansions in the C9ORF72 locus that increase risk for both amyotrophic lateral sclerosis and fronto-temporal dementia. C9ORF72 repeat expansion have also been identified in a low percentage of cases with Alzheimer diagnosis (Harms et al., 2013) and in cases with atypical Parkinsonism (Wilke et al., 2016) and in cases with corticobasal syndrome.

Detailed analysis of histopathology and molecular defects in neurodegenerative disease also reveal that there are overlaps between the features of different neurodegenerative diseases.

Extensive work continues to be carried out on the mechanisms of neurodegeneration in the hope that insights into mechanisms may promote development of disease altering therapies.

Underlying manifestations include degeneration of neurons, cellular damage, and generation of waste products that overwhelm disposal mechanisms. Properties of the aggregated protein include prion like properties including self-replication and cell to cell transmission.

### Alzheimer disease: variants in early onset and late onset forms

Genes in which specific variants play a major role in early onset forms of Alzheimer disease may perhaps only play a minor role in late onset forms of the disease. Presenilin variants have been shown to play a major role in early onset forms of the disease. Defects in presenilin function lead to abnormal processing of beta amyloid. In some cases of early onset Alzheimer disease duplication in the amyloid precursor gene have been identified.

Thus far there have been no conclusive links established between presence of beta amyloid aggregates in the brain and development of cognitive decline.

There is some evidence that Tau deposits may be related to cognitive impairment (Cho et al., 2016). There is evidence that by the time cognitive symptoms develop, neuronal loss has occurred.

### Other risk factors in Alzheimer disease

Other cellular processes reported to be involved in Alzheimer disease include DNA repair defects, mitochondrial functional impairments, and defective calcium homeostasis (Ridge et al., 2016).

The presence of a specific genetic variant in the Apolipoprotein E gene (APOE) leading to the APOE4 protein, is a well demonstrated risk factor in Alzheimer disease (Mahley, 2016). The mechanistic impact of APOE4 is in part related to the fact that the specific amino acid change leads to altered interactions between the domains within the protein. APOE is a constituent of lipoprotein synthesized primarily in the liver. It is also synthesized in the brain by oligodendrocytes and neurons. Mahley reported that APOE protein is secreted in larger quantities by injured neurons. Mahley (2016) reported the APOE4 drives accumulation of amyloid beta 42 and it drives hypophosphorylation of tau in the hippocampus. He postulated further that impairment of synaptic function and cognitive impairments are driven by impaired lipidation of APOE4. A number of studies have revealed that the APOE4 protein is hypolipidated compared to the other APOE allelic forms.

Key to APOE function is its lipidation. Lipidation of APOE is dependent upon ATP binding cassette transporters including the product of the ABCA1 gene. Koldamova et al. (2014) emphasized that ABCA1 plays a key role in the functionality of APOE. The ABCA1 protein plays important roles in adding lipid molecules to apolipoproteins APOA1 and to APOE. Common and rare variants of ABCA1 have been shown to influence the risk of Alzheimer disease. Studies have revealed that lipidation of APOE may modulate amyloid beta deposition and clearance.

Koldamova et al. (2014) reported that transcription of the ABCA1 gene is regulated by a number of nuclear receptors including the Liver X nuclear receptor and Retinoic X receptor and the peroxisome proliferation activator receptors PPAR. They reported that ABCA1 is normally expressed in all brain cells.

Studies in mice revealed that when ABCA1 is not expressed, levels of the high density lipoprotein (HDL) are low. HDL plays a key role in reverse cholesterol transport that is involved in the removal of cholesterol from cells and its transport to the liver (Oram and Vaughan, 2000).

Studies on mice have revealed that increased expression of ABCA1 can promote lipidation of APOE4. One factor that stimulates ABCA1 synthesis in mice is bexarotene. However, in humans bexarotene impacts a number of different targets in addition to ABCA1. Boehm-Cagan et al. (2016) reported evidence that another compound CS6253 that increases ABCA1 function is a promising for the treatment of APOE4 related Alzheimer disease. CS6253 was reported as a compound that modulates ABCA1 and cholesterol efflux.

It is important to note that there are genetic variants that increase risk of Alzheimer disease and variants that apparently protect.

## Large scale population sequencing

### Whole genome sequencing

In order to get a perspective on the diagnostic importance of sequencing data it is important to take into account results of population sequencing studies e.g., the 1,000 genomes project. Results for this project actually reported data on low pass genomic sequencing and deep exome sequencing on 2,504 individuals from 26 different populations (Auton et al., 2015). They reported that a specific individual genome comprises ~3 billion base pairs and the genome of a typical individual differs from the reference genome at 4.1 to 5 million nucleotide sites. In a typical individual genome between 1 and 4% of genomic variants (40,000–200,000) had a population frequency <0.5%.

It is particularly important to note that per individual genome ~2,000 variants were identified that had previously been associated with complex common disease. In each individual genome 24–30 variants occurred that were listed in ClinVar as being implicated in genetic diseases.

Different populations differed in the extent to which individual genomes differed from the reference genes and the greatest extent of variation was found in African American populations. Common variants were often shared across populations while rare variants tended to be shared only in closely related populations.

Within the total sample of 2,504 individuals 762,000 variants were rare in the global population, occurring at a frequency <0.5% but among these specific variants occurred with a frequency >0.5% in at least one population.

Investigators also carried out studies to predict the functional significance of variants. They predicted that in a typical genome 149–182 variants would be protein truncating and 10,000–12,000 variants would alter peptides. In addition, ~500,000 variants could potentially alter gene regulation since they occurred in promoter or in insulators, in enhancers and in transcription factor binding sites.

### Structural variations in population genomic sequencing studies

In a report by Sudmant et al. (2015) on structural variation in 2,504 human genomes analyzed following short read whole genome sequencing, investigators estimated that each individual genome had on average 18.4 megabases of structural variation and the included 11.2 megabases of copy number variants. A typical individual genome was reported to contain between 2,100 and 2,500 structural variants including copy number variants and large repeat sequence variants, including insertions deletions and inversions. Through identifying homozygous deletions in specific genomic regions, they identified 200 non-essential genes in human. These were predominantly in immunoglobulin genes.

Zarrei et al. (2015) generated a copy number variant map of the human genome. They updated the Database of Genomic Variants (DGV) a database that includes information on benign and pathogenic structural genomic variants, http://dgv.tcag.ca/dgv/app/home. This database is generated on the basis of 55 different peer reviewed studies on the analysis of copy number variant from oligonucleotide or SNP microarrays and cytogenetic studies. Zarrei et al. emphasized that the map they generated has clinical application. In the updated DGV map they documented 935 medically relevant genes and indicated CNVs that overlapped any of these genes.

The Decipher database https://decipher.sanger.ac.uk/ provides information on copy number variants in specific genomic regions. Structural variants are linked to specific genes they encompass and are classified as pathogenic or not pathogenic. Pathogenic variants are linked to specific information on the phenotype of the individual who carried that variant.

## Precision and personalized medicine

The goals of the precision medicine initiative are to improve diagnosis and to enhance possibilities for preventive medicine and also to improve capabilities for disease directed therapy (Collins and Varmus, 2015).

From the standpoint of genetics and genomics, it will be important to derive in depth knowledge of genetic variation in different population and to appropriately connect genetic variation with phenotypic effects.

Personalized medicine in complex diseases rests on determining which particular risk factor, among the many potential risk factors, is likely to be most important in a particular individual. The next step will be to determine if there are specific therapeutic measures that are relevant to specific risk factors.

In addition, in cases where specific genes are known to cause disease it will be important to understand the mechanism through which specific mutations lead to disease. Ashley (2016) drew attention to the depth of understanding of disease mechanisms required to approach successful treatment of cystic fibrosis. One specific mutation in the CFTR gene that leads to disease is the G551D variant that causes the CFTR encoded conductance channel on the cell surface to fail to open appropriately. This defective function was shown to be corrected by the drug, Ivacaftor (Ramsey et al., 2011). However, the most common CFTR protein mutation that leads to disease is F508del and this mutation causes the channel protein to be misfolded and it does not reach the cell surface. The drug Lumacaftor was shown to improve CFTR F508del protein folding. Clinical trials are now in place that use combined treatment with Ivacaftor and Lumacaftor to ensure passage of CFTRf508del to the cell surface and Ivacaftor to facilitate CFTR channel protein on the cell surface (Rehman et al., 2015).

## Conclusion

Advances in nucleic acid sequencing techniques and comprehensive sequencing analyses in different populations, have revealed the surprising degree of individual variation in the human genome. The challenge now is to determine which of the genomic variants are involved in disease causation. Progress in these tasks is being facilitated through documentation of genomic variants, including sequence variants and structural genomic variants, in comprehensive databases. In addition, there are ongoing and expanding efforts to carefully document genotype phenotype correlations and to analyze the biochemical and functional implications of specific sequence variants.

Discoveries of genomic sequence variants have greatly expanded possibilities for accurate molecular diagnosis of genetically determined diseases and have in a growing number of cases, opened the way for targeted therapies. DNA analysis of tumors and analysis of aberrant proteins in cancer have facilitated design of target therapies and approaches to immunotherapy of cancers.

## Author contributions

The author confirms being the sole contributor of this work and approved it for publication.

### Conflict of interest statement

The author declares that the research was conducted in the absence of any commercial or financial relationships that could be construed as a potential conflict of interest.
